# Linking molecular architecture to antibacterial functions: a chemical biology view of cyclic depsipeptides

**DOI:** 10.3389/fchem.2026.1790990

**Published:** 2026-05-08

**Authors:** Sachin Baravkar, Varsha J. Thombare, Yuezhou Wu, Nitin A. Patil

**Affiliations:** 1 Center for Drug Discovery, RTI International, Research Triangle Park, Durham, NC, United States; 2 Biomedicine Discovery Institute, Monash University, Clayton, VIC, Australia

**Keywords:** antimicrobial drugs, cyclic peptide, depsipeptides, peptide synthesis, structure-activity relationship (SAR)

## Abstract

Antimicrobial depsipeptides and cyclodepsipeptides represent a structurally diverse class of natural products that have gained renewed attention as both antibacterial agents and molecular tools to interrogate bacterial physiology. Characterized by the incorporation of ester linkages within macrocyclic peptide frameworks, these molecules exhibit unique conformational properties that underpin distinct modes of action including lipid II recognition, membrane perturbation, and enzyme modulation. This review summarizes advances in discovery, chemical synthesis, and structure activity relationship studies across major depsipeptide families, highlighting how precise chemical features such as macrocycle topology, stereochemical configuration, hydroxylation patterns, and lipidation dictate biological function. By integrating mechanistic insights from chemical biology, microbiology, and medicinal chemistry, we illustrate how cyclodepsipeptides serve as privileged scaffolds for probing bacterial cell envelope organization and essential biosynthetic pathways. In the context of escalating antimicrobial resistance and limited innovation in small molecule antibiotics, these macrocyclic peptides offer valuable opportunities for mechanism driven probe development and therapeutic translation. Collectively, this article provides a unified framework linking molecular structure to antibacterial activity and aims to guide the rational design of next-generation peptide-based antibiotics.

## Introduction

The escalating prevalence of resistant bacterial strains has arisen as a significant challenge to the global healthcare system (Sengupta et al., 2013; Ventola, 2015). Multidrug-resistant bacteria strains such as *Staphylococcus aureus*, *Klebsiella pneumoniae*, *Acinetobacter baumannii*, *Pseudomonas aeruginosa*, and *Bacillus subtilis* have emerged as particularly concerning due to their increasing resistance to current available antibiotics (Zhen et al., 2019). This growing resistance highlights the critical need for developing new antibacterial agents that operate through novel mechanisms of action.

To combat the impending threat of antimicrobial resistance significant efforts have been directed toward the development of both small-molecule antibiotics and antimicrobial peptides (AMPs). Although small molecules have traditionally dominated antibiotic discovery, AMPs offer a compelling alternative due to their broad-spectrum activity and reduced likelihood of resistance development (Xuan et al., 2023). Amongst the AMPs, naturally occurring depsipeptides form the predominant class as they can be found in a wide range of organisms, including bacteria, fungi, plants, algae, sponges, and marine organisms (Zeng et al., 2023). Depsipeptides are peptides where one or more amide bonds are replaced with ester bonds, increasing lipophilicity and enhancing cell permeability, making them valuable in drug development and research (Lemmens-Gruber et al., 2009). Though linear depsipeptides do exist, most of the naturally occurring depsipeptides are cyclic in nature and these cyclodepsipeptides (CDPs) exhibit a broad spectrum of biological activities including antitumor, antifungal, antiviral, antimalarial, and antitrypanosomal activity (Moore, 1996). Due to their unique structural and biological properties, CDPs have emerged as promising lead structures for crop protection and human and veterinary medicine (Sivanathan and Scherkenbeck, 2014).

**FIGURE 1:**
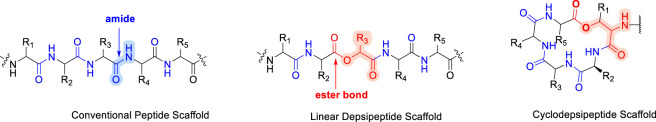
General structural frameworks of peptides, depsipeptides, and cyclodepsipeptides.

The formation of an ester bond in depsipeptides is facilitated through the reaction between a carboxylic acid of one amino acid and side chain hydroxyl group from another amino acid residue. The position of the hydroxy group involved in the ester bond formation determines the classification of CDPs. The hydroxy acid residue may contain an α-hydroxy group, a β-hydroxy group, or a longer chain hydroxy group. Some CDPs possess more than one ester bond in their structure, giving rise to the possibility of combinations of groups, such as α+β hydroxy group, longer chain + α-hydroxy acid, and longer chain + β-hydroxy acid (Figure 1). This review focuses on three different architectural classes based on their molecular topology: (i) head-to-tail cyclodepsipeptides, where *N*-terminus is covalently linked to the *C*-terminus via an ester bond; (ii) branched cyclodepsipeptide, featuring a lipid or peptide tail grafted onto a cyclic head; and (iii) specialized architectures, including homodimeric scaffolds and those containing nonproteinogenic β -hydroxy amino acids or aglycons. For each class, we provide specific examples and synthetic routes via either macrolactamization and macrolactonization strategy, illustrating how these strategies are employed to construct the core scaffolds and preserve antibacterial function. While SAR studies have been reported for a small number of CDPs, such as daptomycin and teixobactin, including through recombinant production of analogues, this review aims to provide a concise perspective. Therefore, we focus on key SAR insights derived from total synthesis and natural structural variation, rather than biosynthetically engineered peptides. In this context, the proposed classification framework offers a systematic approach for examining SAR across the diverse chemical space of CDPs.

## Head-to-tail cyclodepsipeptides

This class of CDP represents the most fundamental architectural motif, featured by a single-loop macrocyclic backbone closed via a covalent bond between the *N*- and *C*-termini. The synthetic design for such CDPs primarily revolves around the strategic selection of the macrocyclization site, *posing* a choice between macrolactamization at a peptide bond or macrolactonization at the ester linkage. While the former often utilizes robust coupling reagents like HATU or PyBOP at sterically unhindered residues, the latter provides a route to preserve the integrity of complex peptide segments but requires specialized activation methods such as the Yamaguchi or Shiina lactonization protocols. The SAR strategies for this class typically focus on stereochemical inversion to tune the molecule’s amphiphilicity (Ciulla and Gelain, 2023). In the following sections, we will examine representative examples of head-to-tail depsipeptides to illustrate how these synthetic strategies and SAR insights are applied to modulate their antibacterial functions.

### Alternaramide

**FIGURE 2:**
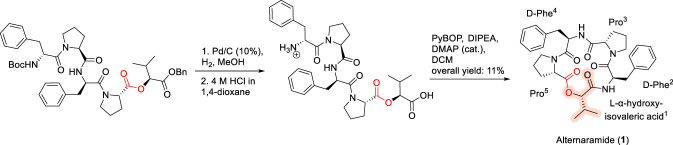
Synthesis of Alternaramide (1) via macrolactamization.

Alternaramide (1) represents a structurally minimalist yet instructive example of how stereochemistry and ring topology influence biological activity. Alternaramide was isolated in 2009 by Oh and co-workers from *Alternaria* species and is characterized as a head-to-tail cyclodepsipeptide composed of four amino acid residues and a single hydroxy acid. The macrocycle incorporates D-phenylalanine residues at positions two and 4, proline residues at positions 3 and 5, and a hydroxy acid at position 1, resulting in a rigid, conformationally constrained framework (Kim et al., 2009). Total synthesis of alternaramide was accomplished via a solution-phase approach in which the hydroxyl functionality was protected as a benzyl ester, followed by hydrogenolytic deprotection and final macrocyclization using PyBOP/DIPEA (Figure 2) (Horton et al., 2011). Notably, this macrolactamization strategy proved superior to macrolactonization under same reaction conditions, delivering the natural product in significantly higher yield and underscoring the importance of cyclization mode in accessing strained CDP architectures. From a biological perspective, alternaramide displays only weak antibacterial activity against *Bacillus subtilis* and *Staphylococcus aureus*, with no detectable activity against *Candida albicans*. Despite this modest profile, extensive SAR studies have provided valuable insights, revealing that the composition and stereochemical configuration of residues within the macrocycle are key determinants of activity. In particular, the presence of D-amino acids was found to be critical, highlighting how stereochemical inversion can profoundly influence bioactivity even within relatively simple cyclodepsipeptide frameworks (Kim et al., 2009). Together, these observations position alternaramide as a useful model for probing the role of stereochemistry and cyclization strategy in modulating the biological properties of small macrocyclic peptides (Flores-Holguín et al., 2021; Kim et al., 2009).

### Valgamicins

**FIGURE 3:**
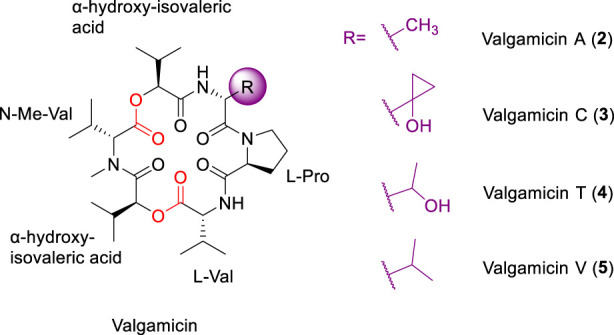
The structure of Valgamicin analogues A, C, T and V.

Valgamicins A, C, T, and V (Figure 3, 2-5) are structurally related hexadepsipeptide macrocycles isolated from *Amycolatopsis species* (Hashizume et al., 2018). These compounds are characterized by a compact macrocyclic scaffold comprising four valine residues, a single proline residue, and a sixth amino acid that varies among the family members and may be either proteinogenic or non-proteinogenic (Hashizume et al., 2018). These compounds are characterized by a compact macrocyclic scaffold comprising four valine or valine mimetic residues, a single proline residue, and a sixth amino acid that varies among the family members and may be either proteinogenic or non-proteinogenic. Notably, valgamicin C (3) incorporates cleonine, a distinctive methylcyclopropanol-containing residue, highlighting the structural diversity tolerated at this position (Hashizume et al., 2018).

Despite their shared core head-to-tail CDP architecture, valgamicins generally lack potent antibacterial activity against Gram-negative pathogens. Limited activity has been reported against Gram-positive bacteria, with valgamicins A (2) and T (4) exhibiting weak inhibitory effects against *Staphylococcus, Enterococcus*, and *Bacillus* species (Hashizume et al., 2018). In contrast to their modest antibacterial profiles, certain valgamicins, particularly 3 and 4, display measurable cytotoxicity against human tumor cell lines. Intriguingly, substantial differences in cytotoxic potency are observed despite close structural similarity, suggesting a sensitive dependence on specific functional groups (Little et al., 2024). In this context, the presence of a hydroxyl group in D-*allo*-threonine in 4 and the cleonine residue in 3 has been proposed to play a critical role in modulating cytotoxic activity (Hashizume et al., 2018). Collectively, these observations underscore the nuanced influence of subtle side-chain modifications on biological outcomes and highlight the need for systematic structure–activity relationship studies to better define the determinants of antibacterial versus cytotoxic activity within the valgamicin family.

### Emericellamide

**FIGURE 4:**
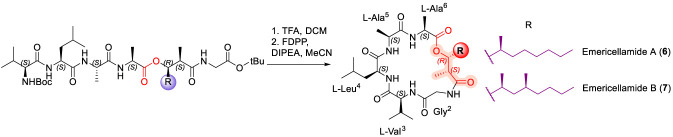
Macrolactamization of Emericellamide A (6) and B (7).

Emericellamides A (6) and B (7) (Figure 4) are 6-mer CDP isolated from the fermentation broths of *Emericella* sp. and *Salinispora arenicola* (Chiang et al., 2008). Both emericellamide A and B exhibit modest antibacterial activity against Gram-positive bacteria, including methicillin-resistant *Staphylococcus aureus* (MRSA) (Li et al., 2009; Oh et al., 2007). The key ester linkage is formed between the *N*-terminal fatty acid, (2R,3R,4S)-3-hydroxy-2,4-dimethyldecanoyl, and the C-terminal alanine residue. The first total synthesis of emericellamide A was reported by Ghosh and co-workers, who achieved the key macrolactamization step using pentafluorophenyl diphenylphosphinate (FDPP) as the coupling reagent (Pradhan et al., 2013). Subsequently, several groups have described alternative synthetic routes to emericellamides A and B employing both solution-phase and solid-phase peptide synthesis strategies (Ma et al., 2009; Priester and Kazmaier, 2023). Consistent with trends observed for other antibacterial CDPs, the modest activity of emericellamides appears to reflect a strong dependence on macrocyclic architecture and amphipathic balance, with limited tolerance for structural perturbation within the depsipeptide ring (Ma et al., 2009).

## Branched cyclodepsipeptides

Branched-CDPs, often described as “lariat-shaped” architectures, represent a more complex topological class characterized by a distinct structural bifurcation. From a chemical biology perspective, the functional hallmark of these molecules is their dual-domain action: the linear branch often facilitates initial membrane partitioning or outer-membrane engagement, while the cyclic core provides the precise spatial orientation required for sequestering cell-wall precursors like Lipid II or inducing depolarization (Ling et al., 2015; Shukla et al., 2022).

The chemical synthesis of these branched scaffolds mainly depends on when the ester bond is installed. There are two common strategies: (i) on-resin esterification to create the ester link first, followed by macrolactamization to close the ring; or (ii) assembling the entire linear protected peptide and then undergoing macrolactonization as the final step (tumescenamide and globomycin). Choosing between these routes is critical to achieving higher yields and avoiding side reactions like epimerization (Ling et al., 2015; Shukla et al., 2022). The following Table 1 summarizes the key SAR strategies for this class, highlighting how modifications to the lipid tail, the ester-to-amide switch, the ring structure, and the substitution of cationic residues influence their antibacterial potency and spectrum.

**TABLE 1:** Examples of modification strategies and quantitative outcomes for branched-CDPs.

Modification strategy	Peptide & specific modification	Target pathogen & biological outcome
Linearization	Laterocidine and Brevicidine: linear amide analog (Ballantine et al., 2023)	Gram- and Gram + pathogens: two-fold less active
	Fusaricidine: linearization (Bionda et al., 2012; Stawikowski and Cudic, 2006)	Fungi bacteria: loss of activity
Ester to amide isosterism	Daptomycin: L-Thr^4^ to L- Dap^4^ (Barnawi et al., 2022; Hart et al., 2014)	Gram + pathogens: 100-fold less active
	Fusaricidins: L-Thr^1^ to L- Dap^1^ (Bionda et al., 2016)	Fungi and Gram + pathogens: maintained activity
	Laterocidine: L-Thr^9^ to L-2,3-Dab^9^ (Al Ayed et al., 2022; Al Ayed et al., 2023; Thombare et al., 2024)	Gram- pathogens: enhanced stability and improved *in vivo* efficacy
	Teixobactin: D-Thr^8^ to D-Dap^8^ (Jad et al., 2015; Jin et al., 2016; Parmar et al., 2016)	*S. aureus*: maintained activity
Lipid tail modification	Fusaricidins: removal of the guanidino group (Bionda et al., 2012)	Fungi pathogens: loss of activity
	Laterocidine: 7-methyloctanoyl to nonanoyl (Ballantine et al., 2022; Ballantine et al., 2023; Thombare et al., 2024)	Gram- pathogens: 8-fold increase in activity
	Viscosins: removal of lipid (Geudens et al., 2017)	Gram + pathogens: loss of activity
	Tumescenamide C: dimethylheptanoyl to acetyl (Takahashi et al., 2020)	*S. Scabiei*: loss of activity
Residue tuning	ADEPs: *N*-methylalanine to pipecolic acid (Hinzen et al., 2006)	Gram + pathogens: enhanced *in vitro* antibacterial activity
	Daptomycin: Orn^6^ and D-Ser^11^ to Ala (alanine scan) (Lam et al., 2013; Lam et al., 2014; Lohani et al., 2015; Moreira et al., 2021)	Gram + pathogens: not critical for activity
	Teixobactin: L-End^10^ to L-Leu/L-Met/L-Chg (Dhara et al., 2016; Jin et al., 2018; Parmar et al., 2017a)	Gram + pathogens: maintained activity or superior
	Globomycin: L-Ser^3^ to Dap^3^ (Inukai et al., 1978; Kogen et al., 2000; Sarabia et al., 2011)	Gram - pathogens: five-fold increase in activity
	Lysobactin: L-β-OHPhe^3^ to L-Thr^3^ (Hall et al., 2012)	Gram + pathogens: maintained activity
N-methylation	Daptomycin: N-methylation of Kyn^13^ (Kynomycin) (Chow et al., 2020a)	Gram + pathogens: improved activity
	Pseudodesmin A: N-methylation of L-Leu^7^ (De Vleeschouwer et al., 2017)	Gram + pathogens: loss of activity

### Teixobactin

**FIGURE 5:**
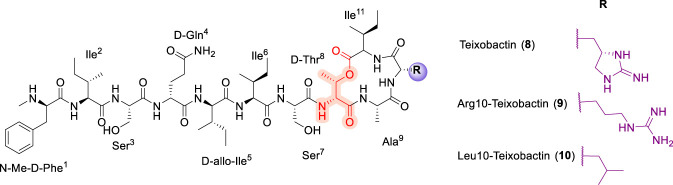
The chemical structure of Teixobactin (8) and its L-*allo*-End analogues (9), (10).

The discovery of teixobactin (TXB, 8) (Figure 5) in 2015 marked a major advance in cyclodepsipeptide antibiotic research and reinforced the therapeutic potential of natural products accessed from previously uncultured microorganisms. Teixobactin was isolated from the β-proteobacterium *Eleftheria terrae* using isolation chip (iChip) technology applied to soil samples collected in Maine (Nichols et al., 2010). Structurally, teixobactin is an 11-residue cyclodepsipeptide comprising seven proteinogenic L-amino acids and four non-canonical D-residues. Two features distinguish this scaffold from other peptide antibiotics discussed above: the presence of the rare guanidinium-containing amino acid L-*allo*-enduracididine (L-*allo*-End) at position 10 and a depsipeptide linkage formed between D-Thr^8^ and L-Ile^11^. Teixobactin displays potent bactericidal activity against a range of Gram-positive pathogens, including methicillin-resistant *Staphylococcus aureus*, *Mycobacterium tuberculosis*, and *Clostridioides difficile*, and notably exhibits a low propensity for resistance development (Jad et al., 2015; Parmar et al., 2016).

Driven by both its exceptional biological profile and the synthetic challenge posed by L-*allo*-End, extensive SAR studies have been undertaken. The first step in the total chemical synthesis of TXB involves preparation of the L-*allo*-End building block, followed by assembly of the depsipeptide chain (Jad et al., 2015; Jin et al., 2016; Parmar et al., 2016). Given that the principal synthetic challenge associated with teixobactin arises from the complexity of the L-*allo*-End residue, early synthetic efforts focused on replacing this moiety with more accessible cationic amino acids. These studies revealed that Arg^10^-TXB (9) retained antibacterial activity but was approximately ten-fold less potent than the native compound. Subsequent analogues incorporating homo-arginine (Parmar et al., 2017b), nor-arginine (Jin et al., 2017), lysine (Wu et al., 2017), ornithine (Abdel Monaim et al., 2017), diaminopropanoic acid (Dap) (Parmar et al., 2017b), diaminobutyric acid (Dab) (Parmar et al., 2017b), or guanidino-substituted residues such as guanidinoaminopropionic acid (GAPA) (Parmar et al., 2017b) further demonstrated that, while a cationic side chain at position 10 is beneficial, it is not sufficient to fully recapitulate native potency. In contrast, replacement with anionic amino acids uniformly abolished activity. More nuanced SAR studies revealed unexpected tolerance at this position: halogenated and alkyl-substituted Arg^10^ analogues exhibited activity comparable to Arg^10^-TXB, while substitution with hydrophobic residues such as Ile, Leu, Val, or Met afforded analogues with potency comparable to or exceeding that of native teixobactin (Jin et al., 2018; Parmar et al., 2017a). Notably, the Chg^10^ analogue displayed low-micromolar activity against *S. aureus* (MRSA) (Parmar et al., 2017a) and Met^10^-TXB analogue also showed equipotent activity with the native TXB (Dhara et al., 2016). Collectively, these SAR studies demonstrate that analogues such as Leu^10^-TXB 10, Met^10^-TXB, and Chg^10^-TXB can match or surpass the activity of teixobactin, indicating that L-*allo*-End, while structurally distinctive, is not essential for antibacterial activity.

Complementary SAR investigations at the *N*-terminus and within the hydrophobic core further delineated the stringent structural requirements of teixobactin. The *N*-terminal N-Me-D-Phe^1^ residue was shown to be critical, as substitution with smaller residues (e.g., Ala) or aliphatic groups such as acetate, benzyl, decyl, dodecyl, or morpholine led to inactive analogues (Jin et al., 2017). Although substitution of N-Me-D-Phe^1^ itself resulted in inactive compounds (Abdel Monaim et al., 2016), the *N*-methyl group was found to be dispensable, and biphenyl replacement at the *N*-terminus afforded analogues with enhanced activity. Teixobactin also contains four isoleucine residues at positions 2, 5, 6, and 11, including one D-*allo*-isoleucine, which together form a hydrophobic domain essential for antibacterial activity. Lysine-scanning and alanine-mutagenesis studies conducted by Albericio and co-workers demonstrated (Abdel Mona et al., 2016) that residues at positions 2, 5, and six are particularly intolerant to substitution, with even conservative changes resulting in marked or complete loss of antibacterial potency (Abdel Mona et al., 2016). Replacement of Ile^2^ with Ala, Phe, Thr, Leu, or Val led to a pronounced reduction in activity (Yang et al., 2016). Similarly, substitution at Ile^6^ with valine or other hydrophobic amino acids resulted in a substantial loss of antibacterial activity. Alanine scanning further confirmed that positions 2, 5, and 6 are poorly tolerated, with alanine substitution at these sites leading to complete loss of activity (Chen et al., 2017). Macrocyclic integrity remained a defining feature of TXB, as lactam analogues preserved activity against *Staphylococcus aureus*. Overall, teixobactin SAR reveals a recurring theme observed across cyclodepsipeptide antibiotics: while peripheral or distinctive residues such as L-*allo*-End can be modified or replaced, preservation of the macrocyclic framework and the spatial organization of key hydrophobic and cationic elements is essential for maintaining potent antibacterial activity.

From a mechanistic standpoint, the emerging SAR landscape of teixobactin closely mirrors principles established for other lipid II–targeting cyclodepsipeptides such as ramoplanin and enduracidin. In all three cases, antibacterial activity is governed less by the presence of any single distinctive residue and more by preservation of a conformationally constrained macrocyclic framework that enables multivalent engagement of conserved cell wall precursors. For teixobactin, extensive SAR studies demonstrate that while the rare L-*allo*-enduracididine at position 10 enhances native potency, it is not strictly required for activity, provided that a suitable hydrophobic or cationic residue maintains the spatial organization necessary for lipid II binding (Ling et al., 2015; Shukla et al., 2022). This tolerance parallels observations in ramoplanin, where glycosylation and peripheral side-chain modifications can be accommodated without loss of activity as long as the macrocyclic depsipeptide ring remains intact, and in enduracidin, where variations in aromatic halogenation exert minimal influence on antibacterial potency (Jad et al., 2015; Parmar et al., 2016). Conversely, disruption of the macrocycle or perturbation of key hydrophobic domains consistently abolishes activity across all three scaffolds. Collectively, these comparisons reinforce a unifying mechanistic paradigm in which rigid macrocyclic architecture and amphipathic surface presentation, rather than precise side-chain identity, dictate productive lipid II recognition (Ling et al., 2015; Shukla et al., 2022). Such insights provide a compelling framework for the rational design of next-generation lipid II–binding antibiotics that balance structural simplification with retention of potent, resistance-resilient antibacterial activity. These SAR and mechanistic insights continue to guide the rational design of simplified yet highly active teixobactin analogues with improved synthetic accessibility.

### Daptomycin

Daptomycin (11) is a 13–amino acid lipo-cyclodepsipeptide produced by the bacterium *Streptomyces roseosporus* and was discovered by researchers at Eli Lilly in 1980 (Debono et al., 1988; Robbel and Marahiel, 2010). Daptomycin exhibits potent antibacterial activity against Gram-positive pathogens, including methicillin-resistant *S. aureus* (MRSA) and vancomycin-resistant enterococci (VRE) (Steenbergen et al., 2005). It exerts its antibacterial effect by disrupting bacterial membrane function, leading to rapid membrane depolarization and subsequent cell death. In contrast to many other antibiotics, daptomycin lacks activity against Gram-negative bacteria, largely due to its inability to penetrate the outer membrane. Clinically, daptomycin is approved for the treatment of complicated skin and soft tissue infections, as well as bloodstream infections (bacteremia) and infective endocarditis (Baltz et al., 2005).

**FIGURE 6:**
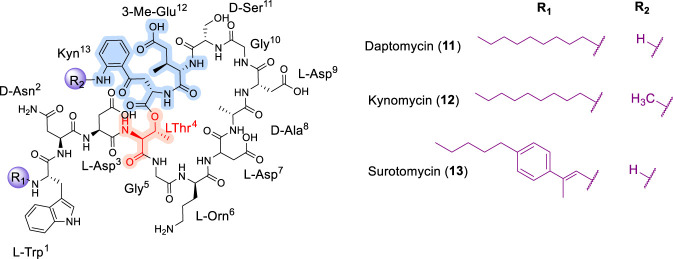
The chemical structure of Daptomycin (11) and analogues Kynomycin (12) and Surotomycin (13).

Structurally, daptomycin comprises three D-configured amino acids (D-Asn^2^, D-Ala^8^, and D-Ser^11^) and several non-proteinogenic residues, including ornithine (L-Orn^6^), (2S,3R)-methyl glutamate (L-3-Me-Glu^12^), and L-kynurenine (L-Kyn^13^) (Figure 6). Cyclization to form the lactone core occurs via an ester bond between the C-terminal carboxyl group of L-Kyn^13^ and the hydroxyl group of L-Thr^4^. The linear tripeptide segment consists of L-Trp^1^, D-Asn^2^, and L-Asp^3^. The *N*-terminus is acylated with an *n*-decanoyl fatty acid chain, which is integral to the ability of daptomycin to associate with and penetrate Gram-positive bacterial membranes (Debono et al., 1988; Lam et al., 2013; Robbel and Marahiel, 2010).

The first total synthesis of daptomycin was reported by Li and co-workers (Lam et al., 2013), who employed a combination of solution-phase and solid-phase peptide synthesis coupled with chemoselective serine ligation. Since then, several groups have developed alternative synthetic approaches integrating solution- and solid-phase methodologies (Lam et al., 2014; Moreira et al., 2021). Lohani et al. (2015) reported the first complete solid-phase synthesis of native daptomycin using an azide as an orthogonal protecting group; the azido functionality was subsequently reduced with trimethylphosphine and followed by coupling to the next amino acid (Lohani et al., 2015). In general, synthetic strategies for daptomycin involve assembly of the lipopeptide backbone via either solution- or solid-phase peptide synthesis, followed by installation of the lipid tail (Lohani et al., 2015). Key challenges include stereochemical control of multiple chiral centers and efficient formation of the CDP ring (Lam et al., 2013).

The SAR of daptomycin has been extensively investigated, revealing that both the amino acid sequence and the lipid tail are critical determinants of antibacterial activity (Chow et al., 2020b). Modifications to either element can significantly affect potency and spectrum of activity. For example, changes in the peptide sequence can alter molecular charge and conformation, thereby influencing membrane binding (Chow et al., 2022), while modifications to the lipid tail can affect solubility and lipophilicity, ultimately impacting membrane penetration. The unique membrane-targeting mechanism of daptomycin also contributes to its potency and broad-spectrum activity against Gram-positive bacteria (Steenbergen et al., 2005).

Scott D. Taylor and co-workers reported a synthetic strategy in which simplified analogues were prepared by replacing L-3-Me-Glu^12^ with glutamate and L-Kyn^13^ with tryptophan (Lohani et al., 2016). In a related study, replacement of the threonine-derived depsipeptide ester bond with a serine-based linkage yielded an analogue that was approximately 20-fold less active than native daptomycin (Lohani et al., 2016). Martin and colleagues subsequently reported a series of daptomycin analogues synthesized using a combined solid- and solution-phase approach (Hart et al., 2014). A key feature of this strategy involved replacing the ester bond between L-Thr^4^ and L-Kyn^13^ with an amide linkage via incorporation of (S)-2,3-diaminopropionic acid (Dap). However, these lactam analogues were approximately 100-fold less active than the parent compound, potentially due to the absence of the β-methyl group of threonine (Barnawi et al., 2022).

An alanine scan performed at the University of Waterloo on a potent daptomycin analogue bearing L-Glu^12^ and L-Trp^13^ substitutions revealed that the side chains of L-Orn^6^ and D-Ser^11^ were not critical for activity (Barnawi et al., 2022). Additional SAR studies indicated that cationic and polar residues are generally tolerated, whereas substitution of anionic, hydrophobic, or aromatic residues is poorly tolerated. N-Methylation studies conducted by Li and co-workers (Chow et al., 2020a) demonstrated that N-Me-Trp^1^ and N-Me-Gly^5^ substitutions improved potency against *Enterococcus* isolates (Chow et al., 2020a). Notably, N-methylation of the L-Kyn^13^ residue resulted in a dramatic enhancement of potency, as confirmed by both *in vitro* and *in vivo* studies.

Kynomycin (12), obtained via N-methylation at the Kyn^13^ residue of daptomycin, exhibited enhanced activity in preliminary studies (Figure 6). *In vitro* and *in vivo* evaluations indicated that kynomycin was more effective in treating MRSA and VRE infections, retained activity against daptomycin-resistant strains, and showed a reduced tendency to induce resistance in *S*. *aureus* (Chow et al., 2020a).

Surotomycin (13) (Figure 6) was identified as a more potent synthetic analogue of daptomycin (Yin et al., 2015). Surotomycin induces depolarization of the *S*. *aureus* cell membrane without significantly increasing membrane permeability, resulting in loss of cell viability with minimal cell lysis (Mascio et al., 2012). Compared with daptomycin, surotomycin exhibited improved *in vitro* activity against *Clostridioides difficile*, including NAP1 strains, with an approximately eight-fold increase in potency.

Comparative SAR studies of daptomycin, teixobactin, and enduracidin reveal shared design principles governing membrane-targeting cyclodepsipeptide antibiotics. In all three cases, antibacterial activity depends primarily on preservation of a rigid macrocyclic architecture that enables effective interaction with bacterial membranes or membrane-anchored lipid II, rather than strict conservation of individual side chains (Barnawi et al., 2022; Chow et al., 2020a). Peripheral modifications, including changes to distinctive residues or aromatic substitutions, are often tolerated provided that the overall amphipathic balance and spatial organization of hydrophobic and cationic elements are maintained (Chow et al., 2020a). In contrast, disruption of macrocyclic integrity or core hydrophobic domains consistently leads to loss of antibacterial potency.

### Viscosin like peptide

**FIGURE 7:**
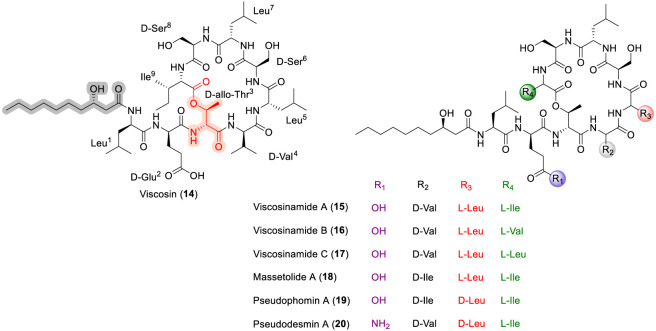
The chemical structure of viscosin family peptide analogues Viscosin (14), Viscosinamide (15–17), Massetolide (18), Pseudophomin A (19) and Pseudodesmin A (20).

Viscosin (14) and its analgoues called as viscosin like peptides, namely, Viscosinamide A-D, Massetolide A-H, pseudodesmin A-B and pseudophomin A-B were isolated from *Pseudomonas fluorescens*, and have been found to exhibit potent antibacterial against Gram-positive bacteria and antifungal activity (Nielsen et al., 1999; Oni et al., 2020; Martin H et al., 2019; Sinnaeve et al., 2009; Bruijn et al., 2008). Viscosinamide A (15) is a 9-mer CDP in which the macrocycle is formed through an ester bond between the hydroxyl group of D-*allo*-threonine at position 3 (D-*allo*-Thr^3^) and the carboxyl group of the C-terminal isoleucine (Ile^9^). Viscosinamide variants A–C differ primarily at position 2, where Glu^2^ is replaced by Gln^2^, and at the C-terminus, which incorporates isoleucine (15), valine (16), or leucine (17) (Figure 7). In comparison, massetolide A (18) contains D-isoleucine at position 4, while pseudophomin A (19) and pseudodesmin A (20) differ at positions 2 and 3 and additionally exhibit stereochemical inversion at Leu^5^.

The synthesis of viscosin-like peptides has been achieved using standard solid-phase peptide synthesis protocols, in which the side-chain hydroxyl group of threonine is employed to form an ester bond with the carboxyl group of Ile, Val, or Leu to generate the depsipeptide macrocycle (De Vleeschouwer et al., 2014; Hiramoto et al., 1969; Hiramoto et al., 1971). Structure–activity relationship studies within the viscosin family have identified pseudodesmin A as exhibiting superior antimicrobial activity. N-Methylation studies revealed that modification at position 7 of pseudodesmin A had a pronounced detrimental effect on biological activity, whereas N-methylation at Leu^1^ had little impact on either bioactivity or conformational stability (De Vleeschouwer et al., 2017). In addition, the presence of a lipid tail has been identified as a key contributor to antibacterial activity within this peptide family, highlighting the importance of amphipathic character for effective membrane interaction (Geudens et al., 2017). Notably, the SAR trends observed within the viscosin family closely parallel those reported for daptomycin, wherein antibacterial potency is strongly influenced by macrocyclic integrity, lipid tail contributions, and the precise balance of amphipathic features rather than strict conservation of individual amino acid identities (De Vleeschouwer et al., 2017).

### Laterocidine, brevicidines and relacidines

**FIGURE 8:**
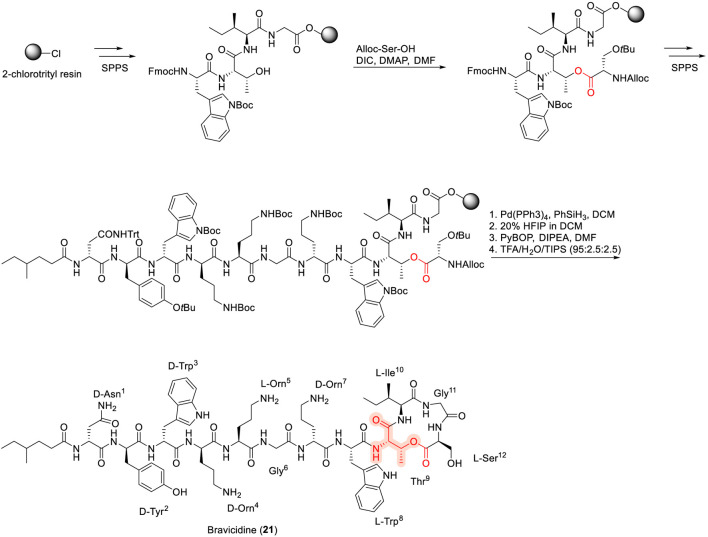
Synthesis of native Brevicidine (21).

Brevicidine (21) and laterocidine (22) (Figure 8) are lipo-cyclodepsipeptides that were first discovered in 2018 from *Brevibacillus laterosporus* DSM 25 and *Paenibacillus alvei* DSM 29, respectively, using genome-mining approaches by Li and co-workers (Li et al., 2018). Both compounds exhibit potent antibacterial activity against a range of Gram-negative pathogens, including *Enterobacter cloacae*, *Escherichia coli*, *Pseudomonas aeruginosa*, *Klebsiella pneumoniae*, and *Acinetobacter baumannii* (Li et al., 2018). Structurally, brevicidine is a 12-residue peptide comprising both proteinogenic and non-proteinogenic amino acids, bearing a 4-methylhexanoic acid lipid tail at the *N*-terminus and a lactone ring formed between the hydroxyl group of Thr^9^ and the carboxyl group of the C-terminal Ser^12^. In contrast, laterocidine is a 13-residue peptide in which macrocyclization occurs through an ester bond between Thr^9^ and Gly^13^. The total syntheses of brevicidine and laterocidine have been reported using hyper–acid-labile 2-chlorotrityl chloride resin, affording overall yields of 10% and 36%, respectively (Hermant et al., 2021).

SAR studies have revealed that brevicidine B (23), an analogue in which Tyr^2^ is replaced by Phe^2^, displays broadened antibacterial activity against both Gram-negative and Gram-positive pathogens (Zhao and Kuipers, 2021). Linear analogues of brevicidine and laterocidine exhibited significantly reduced activity; however, linear amide analogues showed only a two-fold decrease in potency relative to the native compounds (Ballantine et al., 2023). In the same study by Ballantine and co-workers, truncation of Gly^11^ and Ser^12^ in brevicidine, as well as Asn^11^, Gly^12^, and Gly^13^ in laterocidine, did not adversely affect antibacterial activity, whereas removal of the third and fourth residues resulted in a substantial loss of potency. To further define residue-specific contributions, an extended single-residue alanine substitution study (alanine scan) was performed (Ballantine et al., 2022). This analysis revealed that several *N*-terminal residues, including D-Tyr^2^, D-Trp^3^, D-Orn^4^, Orn^5^, D-Orn^7^, and Trp^8^, are critical for antibacterial activity. Substitution at position 9, which serves as the macrocyclization site, was well tolerated with hydrophobic and polar residues, whereas introduction of anionic residues (e.g., Asp) was detrimental. Modification of the lipid tail revealed that replacement with a C10 fatty acid had minimal impact on antibacterial activity (Ballantine et al., 2022; Ballantine et al., 2023). Notably, replacement of Thr^9^ with 2,3-diaminobutyric acid (2,3-Dab i.e., AzaThr) in laterocidine (26) to generate a lactam analogue resulted in enhanced chemical stability and improved *in vivo* efficacy (Al Ayed et al., 2022).

Our pioneering studies on laterocidine further elucidated its SAR and mode of action, highlighting the critical roles of the ester linkage, positively charged residues at positions 4, 5, and 7, and hydrophobic residues at positions two and 3. In particular, the *N*-terminal nonanoyl analogue (27) exhibited an eight-fold increase in antibacterial activity and was shown to act through interaction with the bacterial outer membrane (Thombare et al., 2024). Additionally, a linear brevicidine analogue (28), in which the C10 fatty acid and Asn^1^ were replaced with Orn^1^, demonstrated remarkable antibacterial activity and exhibited excellent biofilm inhibition properties (Yang et al., 2024).

**FIGURE 9:**
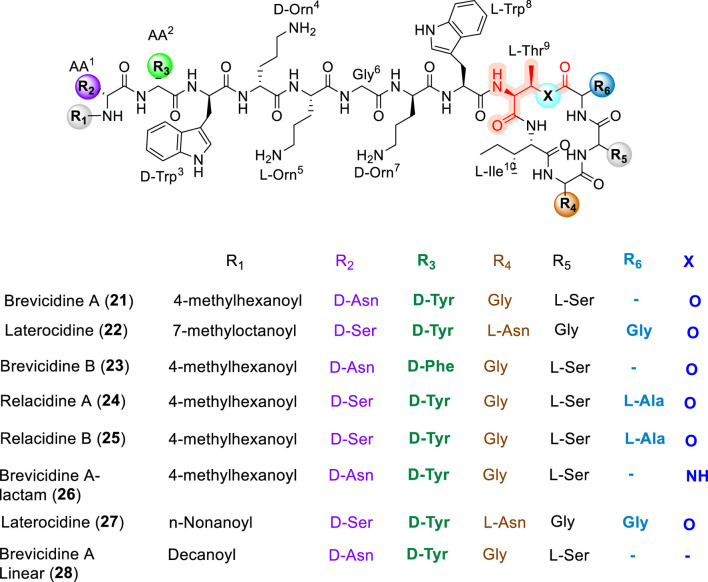
Chemical structures of synthetic analogues of laterocidine and related family peptides, including laterocidine analogues (22, 27), brevicidine analogues (21–23, 26, 28), and relacidine analogues (24–25).

Relacidine A (24) and B (25) (Figure 9) were subsequently isolated and characterized as a class of cationic lipo-cyclodepsipeptides from the soil bacterium *Brevibacillus laterosporus* MG64 (Li et al., 2020a; Li et al., 2020b). Relacidines consist of a 4-methylhexanoic acid lipid tail and a 13-residue peptide backbone, with macrocyclization occurring through a lactone linkage involving the C-terminal residues. These compounds exhibit strong antibacterial activity against Gram-negative bacteria, and mechanistic studies have shown that relacidine B binds to lipopolysaccharides in the bacterial outer membrane (Li et al., 2020b). Karol Al Ayed and co-workers (Al Ayed et al., 2023) reported the total synthesis of relacidines using standard Fmoc/*t*Bu solid-phase peptide synthesis, employing a strategy in which the cyclic core was assembled first by anchoring the hydroxyl group of Ser^12^ to the resin, followed by elongation of the *N*-terminal sequence (Al Ayed et al., 2023). Several diastereomeric relacidine analogues differing at positions 12 and 13 were found to retain equipotent antibacterial activity. Similarly, the lactam analogue relacidamide, in which Thr^9^ was replaced by 2,3-Dab, maintained comparable antibacterial activity while exhibiting improved hemolytic stability (Al Ayed et al., 2023).

Comparative SAR analysis places brevicidine, laterocidine, and relacidine at the intersection of polymyxin- and daptomycin-like design paradigms. Similar to polymyxins, these CDPs rely on a cationic *N*-terminal region and a lipid tail to engage the outer membrane of Gram-negative bacteria, with positively charged residues and lipidation serving as primary determinants of activity. However, unlike polymyxins, which require precise LPS binding motifs and display limited tolerance to macrocyclic modification (Al Ayed et al., 2022; Ballantine et al., 2022; Ballantine et al., 2023; Thombare et al., 2024; Yang et al., 2024). Brevicidine, laterocidine, and relacidine exhibit greater flexibility in macrocycle composition and cyclization chemistry, including tolerance of lactam replacements for ester linkages. In contrast to daptomycin, whose activity is dominated by calcium-dependent membrane depolarization and strict lipid-tail requirements, these scaffolds act through outer-membrane interactions that do not rely on calcium and show broader tolerance to fatty-acid variation (Al Ayed et al., 2022; Ballantine et al., 2022; Thombare et al., 2024). Collectively, these features define a distinct SAR class that combines polymyxin-like outer-membrane engagement with enhanced structural adaptability and reduced reliance on membrane depolarization, offering promising avenues for the development of next-generation Gram-negative antibiotics (Thombare et al., 2024).

### Fusaricidins

**FIGURE 10:**
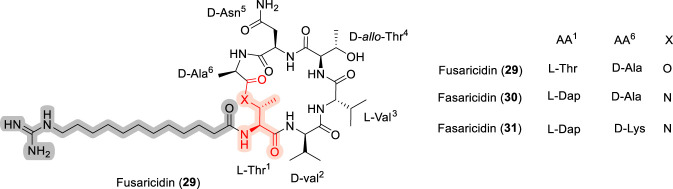
Structure of Fusaricidin (29), Fusaricidin (30) and its D-Lys analogue (31).

Fusaricidins are antifungal cyclodepsipeptides isolated from *Paenibacillus* species and represent a distinct class of antibiotics compared with classical cationic antimicrobial peptides, as they possess only a net +1 charge arising from a single guanidino group located at the terminus of the lipid tail (Kajimura and Kaneda, 1996; Kurusu et al., 1987). Among this family, fusaricidin A (29, Figure 10) is the most potent analogue and features a six–amino acid macrocyclic scaffold in which the depsipeptide bond is formed between the hydroxyl group of Thr^1^ and the carboxyl group of D-Ala^6^. The total synthesis of fusaricidins was accomplished by Cudic and co-workers (Stawikowski and Cudic, 2006) using Fmoc-based solid-phase peptide synthesis strategies (Bionda et al., 2012; Stawikowski and Cudic, 2006). SAR studies revealed that the 12-guanidinododecanoic acid lipid tail, together with hydrophobic amino acid residues, is critical for bioactivity, as modifications to the guanidino-containing terminus significantly influenced antifungal potency (Bionda et al., 2013). While the linear depsipeptide analogue was inactive, the corresponding lactam analogue (30), generated via substitution with diaminopropionic acid (Dap), retained equipotent activity (Bionda et al., 2016). Further refinement of the Dap-containing analogue 30 through a combinatorial chemistry approach led to the development of lactam analogue 31, which effectively inhibited biofilm formation and was capable of completely eradicating mature biofilms formed by both MRSA and *Pseudomonas aeruginosa* (Bionda et al., 2016). In comparative SAR terms, fusaricidins share with brevicidine and laterocidine a reliance on a lipidated scaffold and a terminal guanidinium functionality for productive interaction with bacterial membranes, yet they are distinguished by a lower overall cationic charge and greater tolerance to macrocycle and linkage modification, which may contribute to their unique antifungal and antibiofilm profiles (Bionda et al., 2016).

### Acyldepsipeptide (ADEP) and enopeptin

**FIGURE 11:**
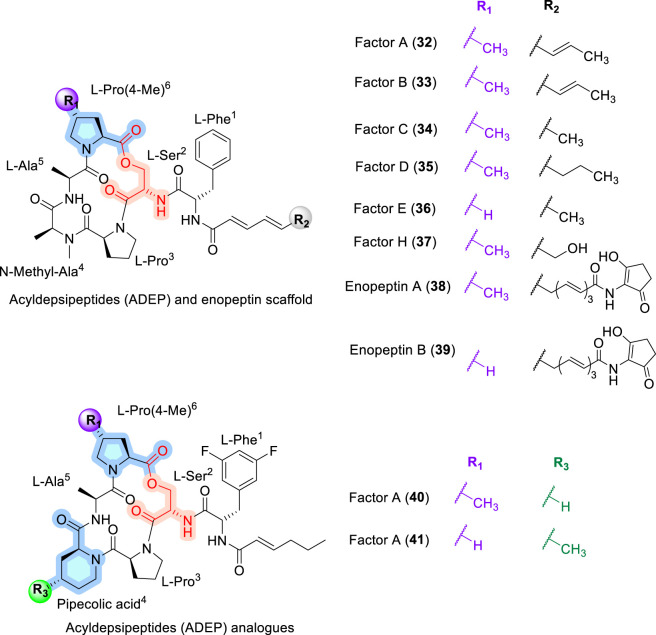
Structure of ADEPs A54556 factors (32–37), enopeptin A-B (38–39) and ADEP analogues (40–41).

Acyldepsipeptides (ADEPs), also known as A54556 factors A–H (32–37, Figure 11), are CDPs derived from *Streptomyces hawaiiensis* NRRL 15010 and exhibit notable antibacterial activity against Gram-positive bacteria (Michel et al., 1985). Structurally related CDPs, termed enopeptins A and B (38–39, Figure 13), were subsequently isolated in 1991 from *Streptomyces* sp. RK-1051 (Koshino et al., 1991). These depsipeptides share a common structural motif comprising a cyclic pentapeptide core containing proline, 4-methylproline, alanine, N-methylated alanine, and serine, with macrocyclization achieved via an ester bond between the C-terminal proline carboxyl group and the serine hydroxyl group. In addition, the N-acyl side chain is appended through a phenylalanine residue and extends into a trans-diene or trans-triene moiety (Michel et al., 1985).

ADEPs have attracted considerable attention due to their unique mechanism of action, whereby they activate the caseinolytic protease ClpP, a member of a large family of bacterial serine protease complexes, leading to uncontrolled proteolysis and bacterial cell death (Sass et al., 2011). The total synthesis of ADEPs was first reported by Goodreid and co-workers using a solution-phase peptide coupling strategy (Goodreid et al., 2014). Subsequent structure–activity relationship studies identified several promising analogues, including derivatives incorporating pipecolic acid in place of N-methylalanine and difluorophenylalanine substitutions (40), which demonstrated enhanced *in vitro* antibacterial activity by promoting conformations compatible with ClpP binding (Hinzen et al., 2006). Notably, an *allo*-threonine–containing ADEP analogue (41), reported by Sello and co-workers (Carney et al., 2014). at Brown University, exhibited an approximately 1200-fold increase in antibacterial potency relative to earlier analogues (Carney et al., 2014). In contrast to lipid II–targeting CDPs such as ramoplanin and teixobactin, as well as membrane-active lipopeptides like daptomycin, ADEPs exert their antibacterial activity through enzyme dysregulation rather than membrane interaction or cell-wall precursor sequestration, highlighting a distinct SAR paradigm in which ClpP activation and conformational compatibility dominate SARs.

### Tumescenamide

**FIGURE 12:**
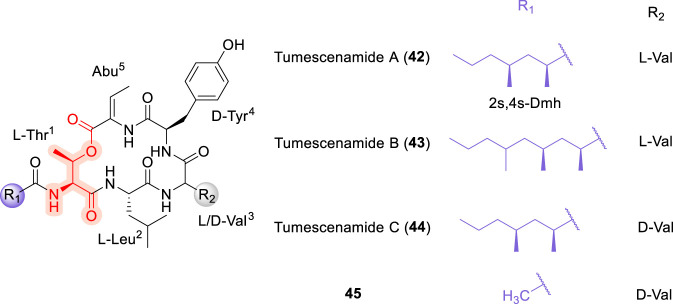
The chemical structure of Tumescenamide A, B and C, and its analogue (45).

Tumescenamides A and B (42, 43; Figure 12) were isolated from *Streptomyces tumescens* YM23-20 (Motohashi et al., 2010), while tumescenamide C (44) was subsequently isolated from the culture broth of an actinomycete *Streptomyces* species (Kishimoto et al., 2012). Tumescenamides are cyclodepsipeptides featuring a lactone linkage formed between the hydroxyl group of L-Thr^1^ and 2-amino-2-butenoic acid (Abu^5^), with the *N*-terminus acylated by a fatty acid moiety, 2,4-dimethylheptanoate (Dmh). Tumescenamide C exhibits antimicrobial activity against Gram-positive bacteria, including *Streptomyces coelicolor* and *Streptomyces lividans*, although its precise mechanism of action remains unclear. In contrast, tumescenamide A has been shown to activate reporter gene expression via the insulin-degrading enzyme promoter, highlighting its potential relevance as a lead compound for Alzheimer’s disease therapy (Motohashi et al., 2010). The first total synthesis of tumescenamide A was reported by Xue et al., which established its stereochemistry and introduced the olefinic functionality through β-elimination of threonine using nano-K_2_CO_3_ (Xue et al., 2022).

**FIGURE 13:**
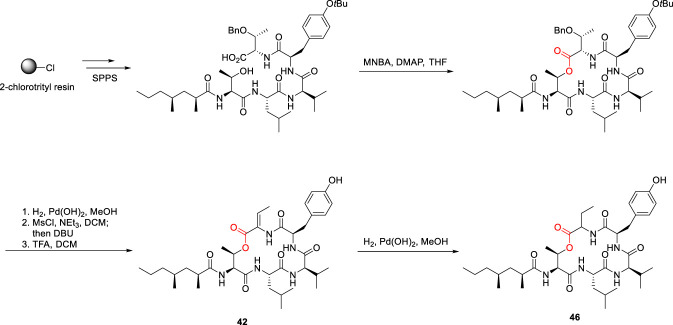
Synthesis of Tumescenamide C, and its analogue 46.

In 2020, the total synthesis of Tumescenamide C was reported by Hideaki Kakeya (Takahashi et al., 2020) (Figure 13), with a primary reliance on Fmoc solid-phase peptide synthesis (SPPS). In this approach, macrolactonization was achieved using 2-methyl-6-nitrobenzoic anhydride (MNBA) as the coupling reagent. Introduction of the olefinic bond was accomplished via mesylation of the threonine hydroxyl group followed by β-elimination using DBU (Takahashi et al., 2020). The acetylated (45) and reduced (46) analogues of tumescenamide C were also synthesized; however, both were devoid of antimicrobial activity, indicating that the Dmh lipid unit and the unsaturation within the Abu residue are critical for bioactivity. The antimicrobial activity of tumescenamide C appears to rely on the appropriate balance of hydrophobicity and size, along with the presence of the α, β-unsaturated carbonyl in the Abu residue. While the hydrophobic moiety likely facilitates activity through enhanced membrane interaction or permeability, the α,β-unsaturated carbonyl may act as a Michael acceptor, thereby contributing directly to antimicrobial efficacy (Takahashi et al., 2020). Notably, the SAR features observed for tumescenamides are consistent with trends reported for other lipo-cyclodepsipeptides, where antimicrobial activity is governed by the interplay between macrocyclic integrity, lipid tail–mediated hydrophobic interactions and strategically positioned unsaturated or electrophilic functionalities (Kaneko et al., 2024).

### Globomycin

**FIGURE 14:**
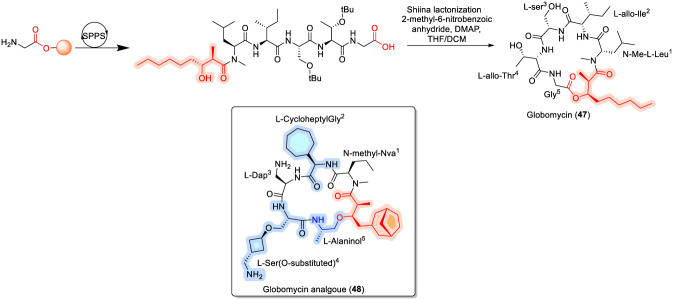
Structure of Globomycin (47), and its analogue (48).

Globomycin (47), produced by *Streptomyces* species, is a selective inhibitor of lipoprotein signal peptidase (LspA) (Inukai et al., 1978; Kiho et al., 2004). This cyclodepsipeptide has demonstrated promising antibacterial activity against members of the Enterobacteriaceae family (Vogeley et al., 2016). Notably, the co-crystal structure of globomycin bound to LspA has provided valuable insights into its binding mode and has opened new opportunities for structure-based drug design (Oppolzer and Lienard, 1993). Structurally, globomycin features an ester linkage formed between the C-terminal Gly^6^ residue and the hydroxyl group of the fatty acid 3-hydroxy-2-methylnonanoic acid. The macrocyclic core comprises N-Me-Leu^1^, L-*allo*-Ile^2^, L-Ser^3^, L-*allo*-Thr^4^, and Gly^5^ (Figure 14). Total syntheses of globomycin have been reported using both solid-phase and solution-phase approaches, with Oppolzer’s sultam chiral auxiliary employed for the stereoselective construction of the lipid fragment (Kogen et al., 2000; Sarabia et al., 2011).

Structure–activity relationship studies have revealed several key determinants of globomycin activity. Ser^3^ was identified as essential, as substitution with alanine resulted in complete loss of activity, whereas introduction of a positively charged residue at this position led to a five-fold improvement in minimum inhibitory concentration (MIC) (Garland et al., 2020). Replacement of L-*allo*-Ile^2^ with aromatic amino acids significantly reduced antibacterial activity, while substitution with aliphatic cyclic residues such as cyclohexylglycine or cycloheptylglycine enhanced potency. Further optimization yielded an active globomycin analogue (48) in which L-*allo*-Ile^2^ was replaced with cycloheptylglycine and L-*allo*-Thr^4^ was substituted with cyclobutylmethanamine-modified serine, resulting in excellent antibacterial activity (Garland et al., 2020). In comparative SAR terms, globomycin differs fundamentally from lipid II–targeting cyclodepsipeptides such as ramoplanin, teixobactin, and lysobactin, as well as from membrane-depolarizing lipopeptides like daptomycin, in that its antibacterial activity is driven by selective enzymatic inhibition of LspA rather than precursor sequestration or membrane disruption, highlighting a distinct and complementary mechanistic niche within the broader CDP landscape (Kogen et al., 2000; Sarabia et al., 2011).

### Lysobactin (Katanosin B)

Lysobactin, a cyclodepsipeptide, was isolated from the fermentation broth of a *Lysobacter* species (ATCC 53042) by scientists at Squibb (Bonner et al., 1988; O'Sullivan et al., 1988). In parallel, Japanese researchers independently identified two closely related natural products, katanosins A and B, from a soil bacterium of the genus *Cytophaga* collected in Katano, Japan (Shoji et al., 1988). Lysobactin (49) and katanosin A (50) exhibit potent antibacterial activity against Gram-positive pathogens, including methicillin-resistant *Staphylococcus aureus* (MRSA) and vancomycin-resistant enterococci (VRE), and have demonstrated promising *in vivo* efficacy in a systemic murine *S. aureus* infection model (Maki et al., 2001). Subsequent mechanistic studies revealed that lysobactin acts by forming a 1:1 complex with Lipid I, Lipid II, and Lipid II_A_^WTA^, key substrates in the peptidoglycan and wall teichoic acid (WTA) biosynthetic pathways (Lee et al., 2016).

**FIGURE 15:**
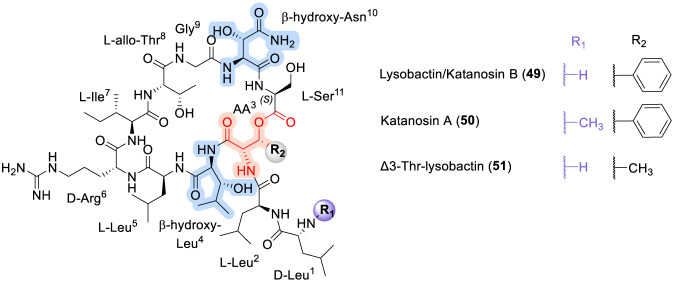
Structure of Lysobactin, Katanosin A (49, 50) and its analogue (51).

Structurally, lysobactin (49) and katanosin A (50) share a common core consisting of a 28-membered macrocycle composed of nine amino acid residues, featuring an ester linkage between the hydroxyl group of a β-hydroxyphenylalanine residue and the carboxyl group of a serine residue (Figure 15). Of the eleven amino acids present in full structure, four are β-hydroxylated variants of proteinogenic residues (von Nussbaum et al., 2007). Total syntheses of lysobactin and katanosin B have been accomplished using both solution-phase and solid-phase peptide synthesis strategies, typically relying on solution-phase preparation of β-hydroxy amino acid building blocks (von Nussbaum et al., 2007; Campagne, 2007; Guzman-Martinez et al., 2007).

Structure–activity relationship studies have further clarified the contributions of individual residues to antibacterial activity. Notably, replacement of the β-hydroxyphenylalanine residue at position 3 with threonine afforded the Δ^3^-Thr-lysobactin analogue (51), which retained high antibacterial potency while exhibiting pronounced membrane-permeabilizing activity (Hall et al., 2012). In comparative SAR terms, lysobactin parallels ramoplanin and teixobactin in its reliance on a rigid, conformationally constrained macrocyclic framework for effective engagement of lipid I and lipid II precursors (von Nussbaum et al., 2007; Campagne, 2007; Guzman-Martinez et al., 2007). However, it is distinguished by its incorporation of multiple β-hydroxylated residues and its ability to simultaneously interfere with peptidoglycan and WTA biosynthesis. In contrast to daptomycin, whose antibacterial activity is driven primarily by calcium-dependent membrane depolarization, lysobactin acts through direct sequestration of lipid-linked cell wall precursors, reinforcing a mechanistic theme shared with other resistance-resilient lipid II–targeting CDPs discussed in this review (Hall et al., 2012).

## CDPS with specialized and sensitive architecture

The final category covers specialized depsipeptide architectures that represent the highest level of structural complexity in this review. Beyond the standard lariat motifs, these molecules feature higher-order complexities, such as the dimeric framework of himastatin, or massive 49-membered macrocycles like those found in ramoplanin and enduracidins. A defining characteristic of this group is the presence of sensitive residues including noncanonical β-hydroxy amino acids and piperazic acid residues, which provide essential rigidity but pose significant challenges for chemical synthesis.

The synthesis of these advanced scaffolds is particularly demanding because of the sensitivity of their functional groups. In particular, the propensity of β-hydroxy residues to undergo side reactions makes both the timing of ester bond formation and the selection of the cyclization site critical. Synthesis often involves: (i) convergent solution-phase assembly, where complex fragments are prepared separately and then coupled; or (ii) specialized solid-phase strategies that use building blocks like Garner’s aldehyde (e.g., skyllamycins) to maintain the chirality of hydroxylated residues. Building on the SAR principles established from simple and branched CDPs, the modification focus here shifts toward scaffold simplification. This involves identifying non-essential elements, such as carbohydrate moieties, specific hydroxyl groups, or peripheral halogens that can be removed to create more synthetically accessible analogues and improve activities. The following Table 2 provides a strategic overview of these simplification efforts, illustrating how complex CDP templates can be streamlined without compromising their potent antibacterial functions.

**TABLE 2:** Examples of modification strategies and quantitative outcomes for complex CDPs.

Modification strategy	Peptide & specific modification	Target pathogen & biological outcome
Monomerization	Himastatin: removal of C5–C5’ biaryl linkage (Sivanathan and Scherkenbeck, 2014; D’Angelo et al., 2022; Kamenecka and Danishefsky, 1998a)	Gram + pathogens: loss of activity
​	Ramoplanin A2: aglycon (removal of carbohydrate) (Marschall et al., 2024; Rew et al., 2004)	Gram + pathogens: two-fold increase in activity
Hydroxyl deletion	Plusbacin A3: removal of β -OH group from Asp (Kim et al., 2013)	Gram + pathogens: loss of activity
	Skyllamycins: removing one or more β -OH group (Giltrap et al., 2018)	Biofilms: moderate to comparable activity retained
Dehalogenation	Enduracidins: removal of Hpg halogens (Yin et al., 2010)	Gram + pathogens: minimal impact on potency
Linearization	Hormaomycins: Change in ring size (Höfer et al., 2011; Reinscheid et al., 2005)	Gram + pathogens: Loss of activity

### Himastatin

**FIGURE 16:**
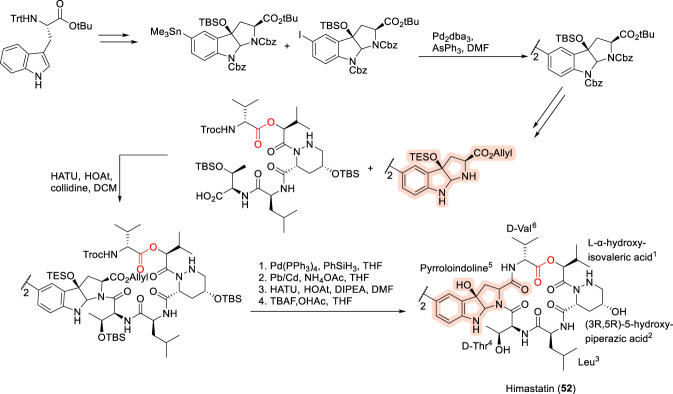
Synthesis of Himastatin (52).

Himastatin (52, Figure 16) represents a structurally sophisticated cyclodepsipeptide that underscores the importance of higher-order architecture in antibacterial activity. Himastatin was originally isolated from *Streptomyces himastatinicus*, discovered in the Himachal Pradesh region of India, and was shown to exhibit potent activity against Gram-positive bacteria as well as antitumor properties (Xie et al., 2019). Structurally, himastatin exists as a symmetric dimer composed of two identical hexapeptide subunits, each incorporating multiple non-proteinogenic residues, including D-valine, L-α-hydroxyisovaleric acid, (3R,5R)-5-hydroxypiperazic acid, D-threonine, and a distinctive tricyclic cyclotryptophan (pyrroloindoline) moiety. The two monomeric units are linked through a central biphenyl framework, with an additional ester bond formed between the hydroxyl group of L-α-hydroxyisovaleric acid and the carboxylate of D-valine. A defining structural hallmark of himastatin is the C5–C5’ covalent linkage between the cyclotryptophan residues, a feature that has been shown to be critical for its potent antibacterial activity against Gram-positive organisms (Sivanathan and Scherkenbeck, 2014; D’Angelo et al., 2022; Kamenecka and Danishefsky, 1998a). The synthetic complexity of this scaffold prompted extensive methodological innovation, culminating in the first total synthesis by Kamenecka and co-workers (Kamenecka and Danishefsky, 1998a; Kamenecka and Danishefsky, 1998b) using a convergent, solution-phase approach (Kamenecka and Danishefsky, 2001). In this strategy, a pentapeptide fragment containing D-valine, L-α-hydroxyisovaleric acid, (3R,5R)-5-hydroxypiperazic acid, L-leucine, and D-threonine was assembled and coupled to a preformed dimeric pyrroloindoline core, which itself was generated via regioselective carbon–carbon bond formation using Stille cross-coupling, followed by macrocyclization to furnish the final dimeric structure (Kamenecka and Danishefsky, 2001). Subsequent structure–activity relationship studies have reinforced the central importance of the biaryl-linked dimeric architecture, demonstrating that preservation of the C–C-linked binuclear motif is essential for maintaining biological activity. At the same time, selective residue modification is tolerated, as exemplified by leucine substitutions that yielded equipotent analogues when replaced with Ser(OMe) or L-Lys(N_3_) (Movassaghi et al., 2024). Collectively, these findings illustrate how himastatin balances architectural rigidity with localized side-chain flexibility, offering valuable insights into how dimeric cyclodepsipeptide scaffolds can be optimized without compromising antibacterial potency.

### Ramoplanin

**FIGURE 17:**
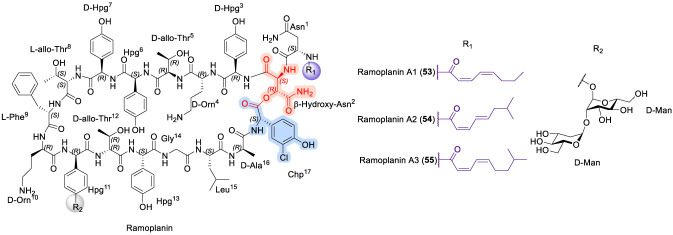
The structure of Ramoplanin analogues A1, A2, and A3.

Ramoplanin (Figure 17) is a lipoglycodepsipeptide antibiotic that was first isolated in 1984 from the fermentation broth of *Actinoplanes* sp. ATCC 33076 (Ma et al., 2009). It exhibits potent antibacterial activity against a broad spectrum of aerobic and anaerobic Gram-positive pathogens by targeting bacterial cell wall biosynthesis (Pallanza et al., 1984). Notably, ramoplanin is reported to be two-to ten-fold more active than vancomycin against several antibiotic-resistant Gram-positive infections. Mechanistically, ramoplanin functions as a competitive inhibitor through high-affinity binding to lipid intermediates I and II, thereby blocking both the intracellular MurG-catalyzed UDP-GlcNAc transfer step and the extracellular transglycosylase-mediated polymerization process (NLM, 1999). This dual-site interference ultimately leads to inhibition of peptidoglycan assembly. Structurally, ramoplanin is produced as a complex mixture of closely related components (A1–A3) (53–55), among which ramoplanin A2 is the principal contributor to antibacterial activity. Ramoplanin A2 (54) has advanced into clinical development and is currently being evaluated in Phase III trials as an oral agent for the treatment of intestinal vancomycin-resistant *Enterococcus faecium* infections, as well as in Phase II trials for nasal decolonization of methicillin-resistant *Staphylococcus aureus* (Cheng et al., 2014; Fulco and Wenzel, 2006; Wong et al., 2001). From a structural standpoint, ramoplanin A2 is a 17-residue peptide featuring a 49-membered macrocyclic lactone formed between the C-terminal 3-chloro-4-hydroxyphenylglycine (Chp^17^) and the β-hydroxyl group of β-hydroxyasparagine at position 2. The synthetic complexity of this scaffold has stimulated extensive methodological development, most notably the convergent solution-phase synthesis reported by Boger and co-workers (Shin et al., 2004). In this work, ramoplanin A2 and a series of analogues were assembled from three strategically designed fragments corresponding to residues 3–9, a pentadepsipeptide segment encompassing residues 1, 2, and 15–17, and a pentapeptide spanning residues 10–14, thereby providing a robust platform for systematic analogue generation and SAR exploration (Jiang et al., 2003; Shin et al., 2004).

**FIGURE 18:**
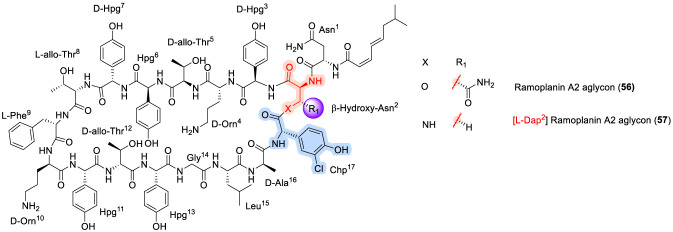
The structure of Ramoplanin analogues A2 aglycon and [L-Dap^2^]A2 aglycon.

Subsequent SAR studies on ramoplanin further clarified the contributions of individual structural elements to antibacterial efficacy. Notably, the aglycon of ramoplanin A2 (55) displayed approximately two-fold greater activity than the parent glycosylated compound (54) against *Staphylococcus aureus* (ATCC 25923), indicating that the carbohydrate moiety is not essential for antibacterial activity (Marschall et al., 2024; Rew et al., 2004). In contrast, hydrolytic opening of the macrocyclic scaffold in both 55 and 56 resulted in complete loss of activity, underscoring the critical role of the intact depsipeptide ring in maintaining bioactive conformation and target engagement (Rew et al., 2004). Efforts to simplify and stabilize the scaffold led to the development of an L-Dap^2^ lactam analogue (57) of the ramoplanin A2 aglycon (Figure 18), which demonstrated a two-to three-fold enhancement in potency relative to both the ramoplanin complex and ramoplanin A2, while also offering improved chemical stability and synthetic tractability (Nam et al., 2007). Further insight into residue-level contributions was gained through alanine-scanning mutagenesis of analogue 57, which identified Orn^4^ and Orn^10^ as key determinants of antibacterial activity (Nam et al., 2007). Overall, these insights reveal how macrocyclic integrity, side-chain functionality, and judicious scaffold simplification collectively govern ramoplanin activity, providing a clear roadmap for the rational development of next-generation analogues with improved potency and translational potential.

### Enduracidins

**FIGURE 19:**
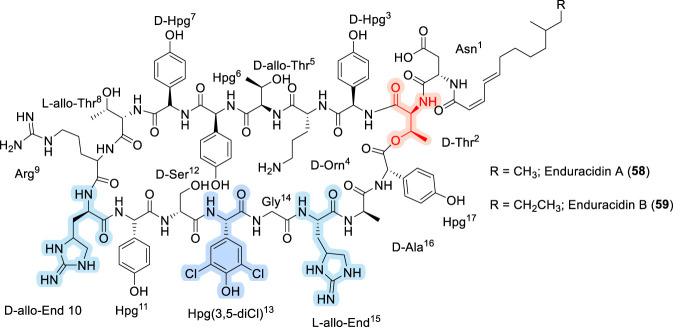
The structure of Enduracidin A and B.

Enduracidins, also known as enramycins, represent another prominent class of lipo-cyclodepsipeptide antibiotics that parallel ramoplanins in both structural complexity and mechanism-relevant SAR features. Enduracidins A (58) and B (59) (Figure 19) are 17-residue lipo-CDPs originally isolated in the late 1960s from fermentations of *Streptomyces fungicidicus* (ATCC21013) (Asai et al., 1968; Higashide et al., 1968). Similar to ramoplanin, enduracidins display potent *in vitro* and *in vivo* activity against a broad range of Gram-positive pathogens, including methicillin-resistant *Staphylococcus aureus* (MRSA) (Komatsuzawa et al., 1994). However, they are distinguished by the presence of a unique (2Z,4E)-branched C12 or C13 fatty acyl chain and a distinct constellation of non-canonical amino acids, including the rare guanidinium-containing residue enduracididine (End), 4-hydroxyphenylglycine (Hpg), 3,5-dichloro-4-hydroxyphenylglycine, citrulline, and ornithine (Asai et al., 1968). These features collectively contribute to a highly constrained, amphipathic scaffold optimized for bacterial envelope targeting. In line with observations made for ramoplanin, early SAR studies on enduracidin emphasize the dominance of the macrocyclic framework and cationic architecture over fine side-chain modifications (Yin et al., 2010). Specifically, preliminary SAR investigations focusing on the halogenation pattern of Hpg residues revealed that variations in chlorination at positions 12 and 13 had minimal impact on antibacterial activity (Yin et al., 2010), suggesting a degree of tolerance at these peripheral aromatic positions. Together, these findings reinforce a recurring theme across lipo-CDP antibiotics, wherein global scaffold integrity and charge distribution are primary determinants of activity, while selective side-chain modifications can be accommodated without compromising antibacterial potency (Yin et al., 2010).

### Plusbacin A3

**FIGURE 20:**
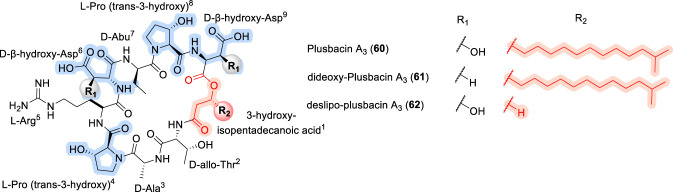
The chemical structure of Plusbacin A3 (60) and its analogues (61, 62).

Plusbacin A3 (60) is a cyclodepsipeptide isolated from *Pseudomonas* species (Maki et al., 2001; Shoji et al., 1992) and exhibits potent antibacterial activity against Gram-positive bacteria, including methicillin-resistant *Staphylococcus aureus* (MRSA) and vancomycin-resistant *Enterococcus faecium* (VRE) (Shoji et al., 1992). Structurally, plusbacin A3 features a lactone linkage formed between an L-threo-β-hydroxyaspartic acid residue and 3-hydroxyisopentadecanoic acid, and incorporates several non-proteinogenic amino acids, including D-threo-β-hydroxyaspartic acid, D-*allo*-Thr, and trans-3-hydroxy-L-proline (Figure 20). Total syntheses of plusbacin A3 have been reported using both solution-phase and solid-phase peptide synthesis (SPPS) strategies (Katsuyama et al., 2017; Katsuyama et al., 2018; Takashina et al., 2022; Wohlrab et al., 2007). SAR studies of plusbacin A3 and its dideoxy analogue (61) demonstrated that the β-hydroxy group of the aspartic acid residue is essential for antimicrobial activity, as its removal results in a pronounced loss of potency (Kim et al., 2013). Likewise, deslipo-plusbacin A3 (62) was found to be inactive, highlighting the critical role of the fatty acid side chain in mediating antibacterial activity (Kim et al., 2013). Notably, these SAR trends closely parallel those observed for emericellamides, where both macrocyclic integrity and lipidation are key determinants of activity, while differing from membrane-depolarizing lipopeptides such as daptomycin and viscosins, in which activity is more strongly governed by amphipathic surface balance and dynamic membrane insertion rather than specific β-hydroxy or lactone functionalities (Katsuyama et al., 2017; Katsuyama et al., 2018; Kim et al., 2013; Takashina et al., 2022).

### Skyllamycin

**FIGURE 21:**
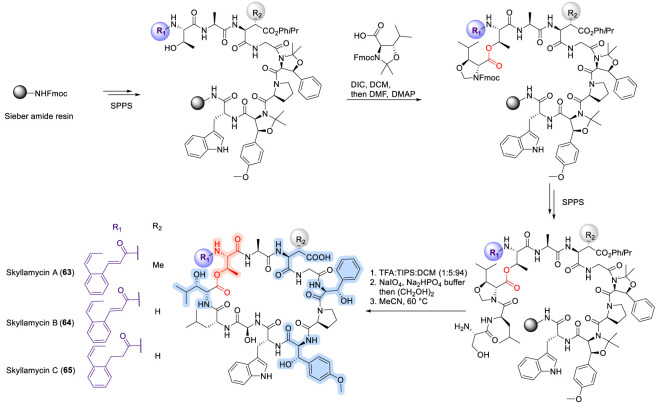
Synthesis of skyllamycin A, B and C.

Skyllamycin A, B, and C are nonribosomal cyclodepsipeptides produced by *Streptomyces* species. Skyllamycin A (63), which exhibits inhibitory activity against the platelet-derived growth factor (PDGF) signaling pathway, was first isolated in 2001 by Matsuda and co-workers (Toki et al., 2001). In contrast, skyllamycins B and C have been shown to inhibit biofilm formation, with skyllamycin B additionally capable of disrupting pre-established biofilms—an activity that is particularly relevant given the role of biofilms in bacterial evasion of antibiotic treatment (Stewart and William Costerton, 2001). Structurally, the skyllamycins possess several unusual features, including three β-hydroxylated amino acids (β-OH-Phe, β-OH-OMe-Tyr, and β-OH-Leu), an *N*-terminal cinnamoyl moiety, β-methylaspartic acid (β-Me-Asp), and the exceptionally rare α-hydroxyglycine (α-OH-Gly) residue (Figure 21).

**FIGURE 22:**
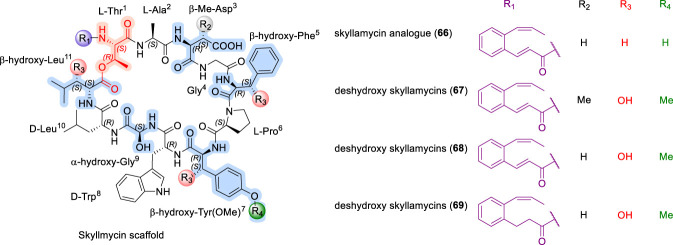
Structure of simplified Skyllamycin analogues (66–69).

The total synthesis of skyllamycin was reported by Payne and co-workers (Giltrap et al., 2017), who employed Garner’s aldehyde as a key intermediate for the preparation of hydroxylated amino acid building blocks, followed by assembly using solid-phase peptide synthesis (Giltrap et al., 2017). In subsequent work, simplified analogues of skyllamycins (66–69, Figure 22) were prepared by selectively removing one or more hydroxyl groups from the corresponding amino acid residues (Giltrap et al., 2018). These analogues retained moderate to comparable biological activity relative to the parent compounds, indicating that such hydroxyl modifications are well tolerated and highlighting opportunities for further structure–activity relationship exploration (Giltrap et al., 2018). Accordingly, the SAR of skyllamycins parallels other biofilm-active CDPs discussed earlier, where macrocyclic integrity and surface functionality are key determinants of activity, yet they are distinguished from earlier families such as daptomycin, ramoplanin and viscosins by their tolerance to extensive hydroxylation and reduced reliance on lipid-driven amphipathic membrane insertion, suggesting a biofilm-focused rather than membrane-disruptive mode of action (Giltrap et al., 2018).

### Svetamycins

**FIGURE 23:**
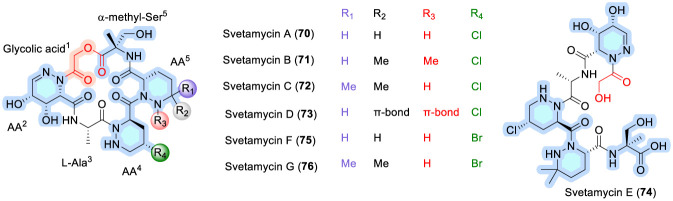
Structure of Svetamycin analogues (70–76).

Svetamycins A–G (70–76, Figure 23) were isolated from a *Streptomyces* species (DSM 14386) and were found to strongly inhibit the growth of *Escherichia coli*, methicillin-resistant *Staphylococcus aureus* (MRSA), and *Mycobacterium smegmatis* (Dardić et al., 2017). Svetamycins A–D (70–74) as well as svetamycins F (75) and G (76) are cyclodepsipeptides, whereas svetamycin E (74) is a linear depsipeptide in which glycolic acid participates in lactone bond formation. Additional distinctive structural features of the svetamycin family include a 4,5-dihydroxy-2,3,4,5-tetrahydropyridazine-3-carboxylic acid residue, a γ-halogenated piperazic acid, and a novel δ-methylated piperazic acid (Dardić et al., 2017; Morshed et al., 2021).

The svetamycins share key SAR features with other piperazic acid–rich CDPs such as hormaomycins and skyllamycins, in which highly functionalized piperazic residues confer conformational rigidity that supports biological activity, while variations in macrocyclization and oxidation patterns modulate antibacterial potency and spectrum. From a mechanistic perspective, svetamycins are thought to operate through non-lytic modes of action that depend on structurally constrained peptide frameworks, thereby distinguishing them from lipid-rich lipopeptides that primarily exert activity through membrane depolarization (Morshed et al., 2021).

### Hormaomycins

**FIGURE 24:**
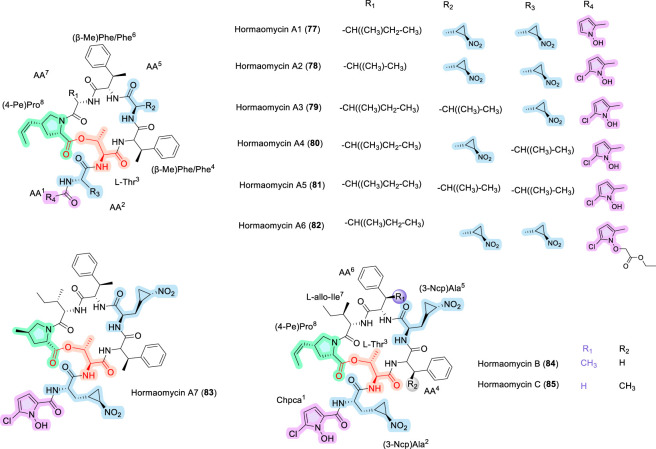
The structure of Hormaomycins A1-A7(77–83), Hormaomycins B (84) and C (85).

**FIGURE 25:**
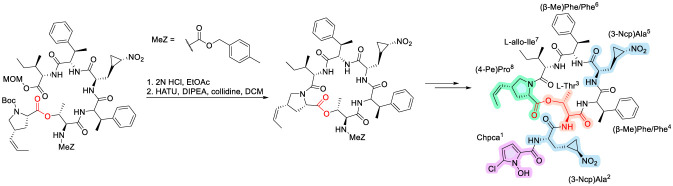
Synthesis of Hormaomycin.

Hormaomycin (Hrm) is a highly modified cyclic depsipeptide natural product, first structurally elucidated in 1990, that was originally isolated from *Streptomyces griseoflavus* W-384 (Andres et al., 1990). Expanding the structural diversity of bioactive cyclodepsipeptides, hormaomycin represents a particularly intricate macrocyclic scaffold that illustrates how fine architectural features can govern antibacterial selectivity and biosynthetic behavior. Its framework contains several rare structural elements, including 3-(trans-2-nitrocyclopropyl)alanine, 4-(Z)-propenylproline, and a chlorinated pyrrole-derived starter unit, making hormaomycin one of the most chemically distinctive peptide natural products described to date (Abdel Monaim et al., 2016; Abdel Monaim et al., 2017). In a key biosynthetic study, Cai and co-workers identified *hrmA* and *hrmB* as positive regulators of hormaomycin production in *S. griseoflavus* W-384 (Cai et al., 2013), where overexpression of *hrmA* and *hrmB* increased production by approximately 40-fold and 135-fold, respectively, and enabled the isolation of six congeners, Hrm A1-A6 (Figure 24) structurally, these included a dechlorinated analogue (Hrm A1, 77), a Val-for-Ile substitution (Hrm A2, 78), and Leu substitutions for one or both 3-NcpAla residues (Hrm A3 -A5 (79–81), whereas Hrm A6 (82) was considered likely to be an extraction artifact. Disruption of *hrmH* did not substantially diminish hormaomycin production, but instead altered product distribution and led to formation of Hrm A7 (83), a likely non-natural congener in which trans-4-methylproline replaces 4-(Z)-propenylproline. Importantly, all seven analogues retained antibacterial activity against *Arthrobacter crystallopoietes*, albeit with differing potencies. Hrm A3 (79) and Hrm A7 (83) displayed activity comparable to native hormaomycin, indicating that certain positions within the scaffold can accommodate substantial modification, whereas Hrm A1 (77) and Hrm A2 (78) were modestly less active and Hrm A4-A6 (80–82) showed markedly reduced potency (Cai et al., 2013). Hormaomycins B (84) and C (85) were discovered in 2015 from a marine mudflat-derived *Streptomyces* sp. SNM55 after changing culture conditions and extending fermentation, an OSMAC-style elicitation approach (Figure 25) (Andres et al., 1990). Members of this family, particularly Hrm B and C, exhibit potent antibacterial activity against Gram-positive pathogens, including *Staphylococcus aureus* and *Staphylococcus pyogenes*, while showing little activity against Gram-negative bacteria (Bae et al., 2015). Mechanistic studies indicate that hormaomycins exert their antibacterial effects through disruption of bacterial membrane potential, ultimately leading to cell death. The pronounced structural complexity of these molecules, which includes unusual residues such as nitrocyclopropylalanine, propenylproline, and β-methylphenylalanine, has posed significant challenges for chemical synthesis (Vanier et al., 2010; Zlatopolskiy and de Meijere, 2004).

Nonetheless, elegant convergent total syntheses have been accomplished by de Meijere and co-workers, enabling systematic interrogation of SAR (Zlatopolskiy and de Meijere, 2004). Key features of these synthetic strategies include careful selection of protecting groups compatible with highly sensitive residues and a critical macrolactamization step achieved under high-dilution conditions using HATU (Vanier et al., 2010; Zlatopolskiy and de Meijere, 2004). The cyclic peptide was then coupled with (3-Ncp)Ala and 5-chloro-1-hydroxypyrrole-2-carboxylic acid to complete the synthesis of hormaomycin (Figure 6). Subsequent SAR studies revealed that the integrity of the cyclic peptide core is indispensable for antibacterial activity, with even subtle alterations in ring size or amino acid composition leading to pronounced losses in potency. The cyclic peptide core of hormaomycins is essential for their activity, as modifications to the amino acid sequence or the size of the cyclic peptide ring can significantly impact on activity (Höfer et al., 2011; Reinscheid et al., 2005). Moreover, the narrow-spectrum activity profile of hormaomycins appears to be strongly influenced by their β-methylphenylalanine residues, which likely contribute to selective membrane interactions in Gram-positive bacteria. Collectively, hormaomycins underscore recurring SAR principles observed across cyclodepsipeptide antibiotics, wherein macrocyclic rigidity and non-canonical side chains act in concert to define both potency and spectrum of activity.

## Summary

Collectively, the CDPs discussed in this review illustrate the remarkable structural and mechanistic diversity through which macrocyclic peptides achieve potent antibacterial activity. Despite their heterogeneity, several unifying SAR principles emerge. Across lipid II–targeting CDPs such as ramoplanin, teixobactin, lysobactin, and enduracidin, preservation of a rigid macrocyclic framework is paramount for high-affinity engagement of conserved cell-wall precursors, while selective tolerance to side-chain modification enables optimization of potency without compromising target recognition. In contrast, membrane-active CDPs and lipopeptides, including daptomycin, viscosins, fusaricidins, brevicidine, and laterocidine, rely on a finely tuned balance of lipidation, amphipathicity, and cationic charge to mediate productive interactions with bacterial membranes or outer-membrane components, with SAR studies consistently demonstrating sensitivity to perturbations in hydrophobic domain organization rather than strict sequence conservation. A distinct third paradigm is exemplified by enzyme-targeting CDPs such as globomycin and acyldepsipeptides, where biological activity is dictated by precise conformational compatibility with protein targets such as LspA or ClpP, rather than membrane engagement or precursor sequestration.

Importantly, subtle scaffold specific features, including ester versus lactam cyclization, beta hydroxylation patterns, piperazic acid incorporation, lipid tail identity, frequently modulate stability, toxicity, and spectrum of activity, underscoring the value of SAR driven scaffold refinement. From a translational perspective, teixobactin and the brevicidine/laterocidine family emerge as particularly promising platforms. Teixobactin combines a resistance-resilient mechanism with broad Gram-positive activity and demonstrated tolerance to significant residue substitution, facilitating synthetic simplification. Meanwhile, brevicidine and laterocidine offer a compelling balance of Gram-negative potency, reduced polymyxin-like toxicity, and exceptional SAR flexibility, including successful lactam analogues with improved stability and *in vivo* efficacy. Together, these scaffolds exemplify how the integration of macrocyclic rigidity with modular chemical adaptability positions CDPs as fertile starting points for the development of next-generation antibiotics capable of addressing both resistance and safety challenges.
